# Minorities Face Delays to Pancreatic Cancer Treatment Regardless of Diagnosis Setting

**DOI:** 10.1245/s10434-024-15352-3

**Published:** 2024-05-24

**Authors:** John Fallon, Oliver Standring, Nandan Vithlani, Lyudmyla Demyan, Manav Shah, Emma Gazzara, Sarah Hartman, Shamsher Pasha, Daniel A. King, Joseph M. Herman, Matthew J. Weiss, Danielle DePeralta, Gary Deutsch

**Affiliations:** 1 Department of Surgical Oncology, Donald and Barbara Zucker School of Medicine at Hofstra/Northwell, Hempstead, NY USA; 2 Department of General Surgery, Northwell Health, New Hyde Park, NY USA; 3 Department of Surgical Oncology, Northwell Health Cancer Institute, Northwell Health, New Hyde Park, NY USA; 4 Division of Medical Oncology/Hematology, Northwell Health, New Hyde Park, NY USA; 5 Department of Radiation Oncology, Northwell Health Cancer Institute, Northwell Health, New Hyde Park, NY USA

**Keywords:** Pancreas cancer, Treatment time, Disparities, Multidisciplinary clinic, Diagnosis location

## Abstract

**Introduction:**

Our analysis was designed to characterize the demographics and disparities between the diagnosis of pancreas cancer during emergency presentation (EP) and the outpatient setting (OP) and to see the impact of our institutions pancreatic multidisciplinary clinic (PMDC) on these disparities.

**Methods:**

Institutional review board-approved retrospective review of our institutional cancer registry and PMDC databases identified patients diagnosed/treated for pancreatic ductal adenocarcinoma between 2014 and 2022. Chi-square tests were used for categorical variables, and one-way ANOVA with a Bonferroni correction was used for continuous variables. Statistical significance was set at *p* < 0.05.

**Results:**

A total of 286 patients met inclusion criteria. Eighty-nine patients (31.1%) were underrepresented minorities (URM). Fifty-seven (64.0%) URMs presented during an EP versus 100 (50.8%) non-URMs (*p* = 0.037). Forty-one (46.1%) URMs were reviewed at PMDC versus 71 (36.0%) non-URMs (*p* = 0.10). No differences in clinical and pathologic stage between the cohorts (*p* = 0.28) were present. URMs took 22 days longer on average to receive treatment (66.5 days vs. 44.8 days, *p* = 0.003) in the EP cohort and 18 days longer in OP cohort (58.0 days vs. 40.5 days, *p* < 0.001) compared with non-URMs. Pancreatic Multidisciplinary Clinic enrollment in EP cohort eliminated the difference in time to treatment between cohorts (48.3 days vs. 37.0 days; *p* = 0.151).

**Results:**

Underrepresented minorities were more likely to be diagnosed via EP and showed delayed times to treatment compared with non-URM counterparts. Our PMDC alleviated some of these observed disparities. Future studies are required to elucidate the specific factors that resulted in these findings and to identify solutions.

Pancreatic ductal adenocarcinoma (PDAC) comprises 2.5% of total cancer diagnoses globally, with the highest age-standardized rates of 8 and 7.8% in North America and Europe, respectively. As of 2022, the 5-year survival rate for pancreatic cancer in the United States remains dismal (5–15%) with a long-term survival rate of only 6%.^1^ Standard cancer-directed treatment approaches can include any combination of chemotherapy, radiation therapy, and surgery.^2,3^ With survival rates continuing to be dismal, focusing on time to treatment has become even more crucial, especially in minority populations.

It has been shown that increased time to treatment in several cancers, including pancreas cancer, leads to an absolute increased risk of mortality ranging from 1.2 to 3.2% per week, highlighting the importance of effective, expeditious navigation of patients into the appropriate care tracts.^4^ Because most patients with PDAC present with a late-stage disease that is ultimately incurable, time to diagnosis and treatment is of the utmost importance.^5^ A recent study determined that initiation of therapy within 6 weeks of diagnosis led to improved survival for clinical stage I and II PDAC patients who underwent resection with curable intent.^6^ The multiple steps required to diagnose and treat PDAC creates several opportunities for delay, which can highlight disparities in the care of certain populations. For PDAC, racial/ethnic disparities have been well-documented in the United States with respect to treatment and survival rates.^7–11^ A 2020 study revealed that non-Hispanic Blacks (NHB) had a significantly worse overall survival compared with non-Hispanic White and Hispanic PDAC patients. However, overall survival for NHB patients was dependent upon income, education, and surgical resection.^7^ Another analysis determined that Black patients were significantly less likely to undergo surgical resection as well as more likely to refuse surgical treatment.^11^ In addition, a non-white race has been shown to significantly decrease the likelihood of receiving stage-appropriate treatment.^9^ Due to the necessity for early detection, diagnosis, and initiation of treatment in PDAC to decrease mortality, it is important to understand the characteristics of patients presenting both in the outpatient setting and during emergent hospital admissions.

This single-institution study was performed at New York’s largest healthcare system, which primarily provides healthcare services to the populations of New York City, Long Island, and Westchester, representing one of the most diverse regions in the world.^12^ The significant population of underrepresented minorities (URM) for which our healthcare institution provides care is ideal for studying disparities in the diagnosis and treatment of various health conditions. The goal of this study was to determine whether there is a difference in the time from initial presentation to diagnosis and treatment for URM patients diagnosed with PDAC compared with non-URM patients in both the inpatient and outpatient settings at our healthcare institution. We also are eager to ascertain whether the implementation of our outpatient multidisciplinary clinic and the subsequent standardization of care reduced any disparities.

## Methods

### Study Population

We performed an institutional review board-approved retrospective review of our institutional cancer registry and pancreatic multidisciplinary clinic (PMDC) databases, identifying patients diagnosed with pancreatic ductal adenocarcinoma via biopsy between 2014 and 2022. Only patients who received definitive treatment were included, defined as receiving any cancer-directed therapeutic intervention (i.e., surgical resection, systemic chemotherapy, radiation therapy). Patients enrolled in our outpatient PMDC were analyzed from 2018 (year PMDC was established) to 2022, whereas non-PMDC patients were evaluated for the entire study period. Patients who presented with symptoms to outside institutions initially, were diagnosed outside of our healthcare system, sought second opinions from outside institutions, or only received second opinions at our institution were excluded.

### Study Variables

Demographic data collected included age, sex, race, ethnicity, location of initial presentation, and comorbidities. Patients were classified as either URM or non-URM based on their self-reported race and ethnicities. African American/Black and Hispanic/Latino patients were defined as URM. White and Asian, non-Hispanic/Latino patients were defined as non-URM. Patients whose race and ethnicity were “other” or “unknown” were excluded from the analysis. Determination of whether patients had a primary care physician (PCP) was determined by the PCP listed in their electronic health record. Date of initial presentation was determined by the date listed in each patient’s chart associated with their first presentation with symptoms leading to the ultimate diagnosis of PDAC; however, in cases where this information could not be obtained, the date of initial abdominal imaging was used as a proxy. Patients who initially presented in the outpatient setting but were sent immediately (same day) to an Emergency Department were considered an emergency presentation (EP). Date of diagnosis was determined by the date of cytologic or histologic confirmation. Date of definitive treatment was determined by extracting the date of first treatment (chemotherapy, radiation therapy, or surgical resection) from patient charts. Patients who underwent surgical resection, but resection was aborted intraoperatively were included, and the attempted surgery date was used as the treatment date. The primary outcome assessed in this retrospective cohort study included the following timepoints: patient presentation date to diagnosis date, diagnosis date to treatment date, and the overall time from patient presentation date to treatment date. Additionally, the diagnosis date will serve as an inflection point throughout our analyses when determining the impact of time from presentation to diagnosis versus time from diagnosis to treatment on overall time from presentation to treatment date.

### Statistical Analysis

Statistical analysis was performed by using SPSS™ 21.0 (IBM Corp, Armonk, NY), and graphs were created by using SPSS™ and Prism™ 7 (GraphPad Software, La Jolla, CA). Binary outcomes compared between two groups were assessed via chi-square analysis with a Bonferroni correction when appropriate, whereas continuous variables were compared for significance with a *t*-test and were reported as mean (± standard deviation). Statistical significance was set at *p* < 0.05.

## Results

### Patient Demographics and Presenting Characteristics

A total of 286 patients met the criteria for analysis in this study. Among these patients, 89 (31.1%) were URM, whereas 197 (68.9%) were non-URM (Table 1). Within the URM cohort, 62 (69.7%) patients were Black, and 27 (30.3%) patients were Latino. In the non-URM group, 168 (85.3%) patients were White, and 29 (14.7%) were Asian. Mean age in the non-URM group was 69.2 (± 10.8) years old and 68.9 (± 10.9) years old in the URM group. No significant differences existed in the gender profiles of each group or in the prevalence of comorbidities, including myocardial infarction, congestive heart failure, hypertension, peripheral vascular disease, cardiovascular disease, dementia, or pulmonary disease. Notably, the URM group had a significantly lower proportion of patients with a primary care physician listed (61.8%, *n* = 55) compared with the non-URM group (76.1%, *n* = 150; *p* = 0.018). Of the URM patients, 46.1% (*n* = 41) were reviewed at the PMDC compared to 36.0% (*n* = 71) patients in the non-URM group. No significant differences existed in the presenting symptoms between the two groups, including pain, jaundice, weight loss, acholic stools, pruritus, and new-onset diabetes. Among clinical and pathological stages of disease at diagnosis, there were no significant differences between the two cohorts, although it should be noted that a plurality of patients in both groups had unknown pathologic stages, primarily due to lack of surgical resection. Despite no differences in symptoms or staging, 64.0% (57) URM patients were more likely to have their disease-presentation at the ED than in the outpatient setting (50.8%, *n* = 100; *p* = 0.037; Table 2).

**Table 1:** Patient demographics

	Underrepresented minorities (URM)	Non-URM	Significance
*n*	31.1% (89)	68.9% (197)	
Age	68.9± 10.9	69.2± 10.8	
Gender			*p* = 0.486
Female	55.2% (48)	47.9% (93)	
Male	44.8% (39)	52.1% (101)	
Ethnicity			
White	–	85.3% (168)	
Asian	–	14.7% (29)	
Latino	30.3% (27)	–	
Black	69.7% (62)	–	
Comorbidities			
Myocardial infarction(MI)	4.5% (4)	2.5% (5)	*p* = 0.380
Congestive heart failure (CHF)	3.4% (3)	2.0% (4)	*p* = 0.497
Hypertension (HTN)	46.1% (41)	40.1% (79)	*p* = 0.344
Peripheral vascular disease (PVD)	2.2% (2)	3.0% (6)	*p* = 0.705
Cardiovascular disease (CVD)	1.1% (1)	2.0% (4)	*p* = 0.588
Dementia	0.0% (0)	0.5% (1)	*p* = 0.501
Pulmonary disease	9.0% (8)	9.6% (19)	*p* = 0.861
Primary care physician (PCP) listed	61.8% (55)	76.1% (150)	***p* = 0.018**

Bolded *p* value indicates statistical significance

Age presented as mean (years) ± SD. All other data presented as %(n)

**Table 2:** Cancer characteristics

	Underrepresented minorities (URM)	Non-URM	Significance
n	89	197	
Reviewed at PMDC	46.1% (41)	36.0% (71)	*p* = 0.180
Presenting Symptoms			
Pain	59.6% (53)	52.8% (104)	*p* = 0.280
Jaundice	37.1% (33)	44.2% (87)	*p* = 0.261
Weight loss	57.3% (51)	49.7% (98)	*p* = 0.236
Endostent	25.8% (23)	31.0% (61)	*p* = 0.379
Acholic Stools	12.4% (11)	14.7% (29)	*p* = 0.594
Pruritus	9.0% (8)	10.7% (21)	*p* = 0.665
New-onset Diabetes	64.0% (57)	61.4% (121)	*p* = 0.672
Location of diagnosis			***p* = 0.037**
ED	64.0% (57)	50.8% (100)	
Outpatient	36.0% (32)	49.2% (97)	
Clinical Stage			*p* = 0.288
I			
A	9.0% (8)	12.7% (25)	
B	22.5% (20)	24.9% (49)	
II			
A	10.1% (9)	7.1% (14)	
B	2.2% (2)	7.1% (14)	
III	12.4% (11)	7.1% (14)	
Unknown	43.8% (39)	41.1% (81)	
Pathological stage			*p* = 0.377
0	7.9% (7)	4.1% (8)	
I			
A	1.1% (1)	4.6% (9)	
B	7.9% (7)	6.6% (13)	
II			
A	4.5% (4)	3.6% (7)	
B	12.4% (11)	17.3% (34)	
III	7.9% (7)	4.6% (9)	
IV	0.0% (0)	2.5% (5)	
Unknown	58.4% (52)	56.9% (112)	

Bolded *p* value indicates statistical significance

All patients who met inclusion criteria and had clinical and or pathological stages in our databases were included in this analysis. Data presented as %(n)

### URM Patients Face Significant and Disproportionate Delays to Treatment in both the ED and Outpatient Settings

When considering all patients in the cohort, URM patients had a significantly higher average time from presentation to diagnosis, diagnosis to treatment, and overall time to treatment (Table 3). The URM (*n* = 89) group on average required 21.2 days to be diagnosed compared with 12.1 days for the non-URM group (*n* = 187; *p* = 0.001) and 36.8 days to receive definitive treatment after being diagnosed compared with 28.4 days for the non-URM group (*p* = 0.022). This led to an overall time to treatment of 58.0 days from initial diagnosis in the URM group compared with 40.5 days for the non-URM group (*p* < 0.001). For the population of patients with an emergency presentation, the overall time from presentation to treatment was significantly longer in the URM group compared with non-URM patients (53.2 days (*n* = 57) vs. 36.3 days (*n* = 100), *p* = 0.004), driven primarily by the increased time from presentation to diagnosis (18.9 days vs. 6.8 days, *p* = 0.001). In contrast, for the population of patients who presented in the outpatient setting, URM patients still had significantly longer total times to treatment (66.5 days (*n* = 32) vs. 44.8 days (*n* = 97), *p* = 0.003), but in these patients the discrepancy was driven primarily by time from diagnosis to treatment (41.2 days vs. 27.3 days, *p* = 0.014).

**Table 3:** Treatment timeline by diagnosis location

	Underrepresented minorities (URM)	Non-URM	Significance
*All patients*			
Time to treatment, Days (Mean)			
Presentation to diagnosis	21.21	12.06	***p* = 0.001**
Diagnosis to treatment	36.76	28.42	***p* = 0.022**
Total time to treatment	57.98	40.48	***p* = 0.000**
Time to treatment, Days (Median)			
Presentation to diagnosis	7.00	7.00	
Diagnosis to treatment	26.00	21.00	
Total time to treatment	44.00	33.00	
*ED-presenting patients*			
Time to treatment, Days (Mean)			
Presentation to diagnosis	18.91	6.78	***p* = 0.001**
Diagnosis to treatment	34.28	29.53	*p* = 0.328
Total time to treatment	53.19	36.31	***p* = 0.004**
Time to treatment, Days (Median)			
Presentation to diagnosis	5.00	3.00	
Diagnosis to treatment	26.00	24.50	
Total time to treatment	39.00	30.00	
*Outpatient-presenting patients*			
Time to treatment, Days (Mean)			
Presentation to Diagnosis	25.31	17.49	*p* = 0.089
Diagnosis to treatment	41.19	27.28	***p* = 0.014**
Total time to treatment	66.50	44.77	***p* = 0.003**
Time to Treatment, Days (Median)			
Presentation to diagnosis	18.00	11.00	
Diagnosis to treatment	27.00	20.00	
Total time to treatment	49.00	37.00	

Bolded *p* values indicate statistical significance

All patients who met inclusion criteria were included in this analysis. Data presented as Days

### URM Discharged Following Emergency Presentation Face Significant and Disproportionate Delays to Biopsy

Of all patients presenting to the Emergency Department, 82.5% were admitted to the hospital with an average length of stay (LOS) of 8 days. Between the URM and non-URM cohorts, there was no significant difference in admission rate (86% vs. 84%) or LOS for those admitted (9.45 days vs. 8.24 days; *p* = 0.436). Overall, no significant difference was found in time-to variables within EP patients between those who were admitted and those who were not. However, upon further stratification we found that within the EP cohort, URM who were discharged from the ED faced significant delays from presentation to biopsy when compared with those admitted through the ED (42 days (*n* = 8) vs. 15 days (*n* = 49); *p* = 0.019). This dramatic discrepancy was not present in the non-URM EP cohort (7 days (*n* = 16) vs. 6 days (*n* = 82); *p* = 0.860; Table 4). Lastly, even when admitted through the ED, URM patients had significantly delayed times to inpatient biopsy and time to overall treatment compared with their non-URM counterparts (15 days (*n* = 49) vs. 7 days (*n* = 82), *p* = 0.023; 50 days vs. 37 days, *p * = 0.048; Table 5).

**Table 4:** Treatment timeline for emergency diagnosis by admission status

	Admitted	Non-Admitted	Significance
*Underrepresented minorities (URM)*			
Time to Treatment, Days (Mean)			
Presentation to diagnosis	15.10	42.25	***p* = 0.019**
Diagnosis to treatment	34.9	30.5	*p* = 0.720
Total time to treatment	50.00	72.75	*p* = 0.140
Time to treatment, Days (Median)			
Presentation to diagnosis	5.00	27.00	
Diagnosis to treatment	26.00	19.00	
Total time to treatment	37.00	46.00	
Presentation to diagnosis			
*Non-URM*			
Time to treatment, Days (Mean)			
Presentation todiagnosis	6.68	7.31	*p* = 0.860
Diagnosis to treatment	30.65	26.25	*p* = 0.564
Total time to treatment	37.33	33.56	*p* = 0.668
Time to treatment, Days (Median)			
Presentation to diagnosis	3.00	8.50	
Diagnosis to treatment	24.50	26.00	
Total time to treatment	29.50	30.50	

Bolded *p* value indicates statistical significance

All patients diagnosed with Pancreatic Ductal Adenocarcinoma following Emergency Presentation were included in this analysis. Data presented as Days

**Table 5:** Treatment timeline for admitted patients following emergency diagnosis

	Underrepresented minorities (URM)	Non-URM	Significance
*Admitted following emergency presentation*			
Time to treatment, Days (Mean)			
Presentation to diagnosis	15.10	6.68	***p* = 0.023**
Diagnosis to treatment	34.9	30.65	*p* = 0.440
Total time to treatment	50.00	37.33	***p* = 0.048**
Time to treatment, Days (Median)			
Presentation to diagnosis	5.00	3.00	
Diagnosis to treatment	26.00	24.50	
Total time to treatment	37.00	29.50	

Bolded *p* values indicate statistical significance

All patients diagnosed with Pancreatic Ductal Adenocarcinoma following Emergency Presentation that were admitted to the hospital were included in this analysis. Data presented as Days

### PMDC Improves Disparities in Time to Care in Select Settings

The association between PMDC enrollment and disparities in treatment were then assessed. Overall, URM patients who were evaluated outside the PMDC faced significantly longer times from presentation to biopsy relative to non-URM patients (Table 6; 25.4 days (*n* = 48) vs. 12.9 days (*n* = 126), *p * = 0.003). Inclusion in the PMDC mitigated this discrepancy (16.3 days (*n* = 41) vs. 10.6 days (*n* = 71), *p * = 0.109), but overall URM patients still had longer overall times from presentation to treatment irrespective of PMDC involvement (64.0 days vs. 41.9 days, *p * = 0.001 in the non-PMDC group compared with 50.9 days vs. 38.0 days, *p * = 0.020 in the PMDC group).

**Table 6:** Treatment timeline by multidisciplinary clinic enrollment

	Underrepresented minorities (URM)	Non-URM	Significance
*Non- pancreatic multidisciplinary clinic (PMDC) patients—all*			
Time to treatment, Days (Mean)			
Presentation to diagnosis	25.42	12.87	***p* = 0.003**
Diagnosis to treatment	38.63	29.03	*p* = 0.071
Total time to treatment	64.04	41.90	***p* = 0.001**
Time to treatment, Days (Median)			
Presentation to diagnosis	8.50	6.50	
Diagnosis to treatment	25.00	20.50	
Total time to treatment	48.50	33.50	
*PMDC patients—all*			
Time to treatment, Days (Mean)			
Presentation to diagnosis	16.29	10.62	*p* = 0.109
Diagnosis to treatment	34.59	27.34	*p* = 0.117
Total time to treatment	50.88	37.96	***p* = 0.020**
Time to treatment, Days (Median)			
Presentation to diagnosis	7.00	7.00	
Diagnosis to treatment	28.00	24.00	
Total time to treatment	42.00	31.00	

Bolded *p* values indicate statistical significance

All patients who met inclusion criteria were included in this analysis. Data presented as Days

Patients were then stratified by presentation setting (ED vs. outpatient) to investigate the impact of the PMDC on time to treatment in these subgroups. Among emergency presentation (EP) patients, URM patients who were not evaluated in the PMDC had longer times from presentation to treatment compared with non-URM patients (Table 7; 57.0 days (*n* = 32) vs. 35.9 days (*n* = 60), *p * = 0.013). Inclusion of the PMDC in the EP cohort eliminated the difference in total time to treatment between these two groups (48.3 days (*n* = 25) vs. 37.0 days (*n* = 40), *p * = 0.151). The elimination of this discrepancy was accounted for primarily by reducing the time from diagnosis to treatment between URM and non-URM patients (37.0 days vs. 28.6 days in the non-PMDC group compared with 30.8 vs. 31.0 days in the PMDC group).

**Table 7:** Treatment timeline for emergency diagnosis by multidisciplinary clinic enrollment

	Underrepresented minorities (URM)	Non-URM	Significance
*Non- pancreatic multidisciplinary clinic (PMDC) patients—emergency Department (ED)*			
Time to treatment, Days (Mean)			
Presentation to diagnosis	20.00	7.27	***p* = 0.016**
Diagnosis to treatment	37.03	28.58	*p* = 0.228
Total time to treatment	57.03	35.85	***p* = 0.013**
Time to treatment, Days (Median)			
Presentation to diagnosis	5.00	2.50	
Diagnosis to treatment	25.50	21.00	
Total time to treatment	42.50	26.00	
*PMDC patients—ED*			
Time to treatment, Days (Mean)			
Presentation to diagnosis	17.52	6.05	***p* = 0.011**
Diagnosis to treatment	30.76	30.95	*p* = 0.977
Total time to treatment	48.28	37.00	*p* = 0.151
Time to treatment, Days (Median)			
Presentation to diagnosis	4.00	3.50	
Diagnosis to treatment	26.00	27.00	
Total time to treatment	39.00	31.00	

Bolded *p* values indicate statistical significance

All patients diagnosed with Pancreatic Ductal Adenocarcinoma following Emergency Presentation were included in this analysis. Data presented as Days

In the outpatient setting, non-PMDC URM patients faced longer times to diagnosis (Table 8; 36.3 days (*n* = 16) vs. 18.0 days (*n* = 66), *p* = 0.007) and total time to treatment (78.1 days vs. 47.4 days, *p* = 0.006) than non-URM patients. Conversely, URM patients who were evaluated in the PMDC did not face longer times to diagnosis (14.4 days (*n* = 16) vs. 16.5 days (*n* = 31), *p* = 0.702). However, there remained a significant difference in total time from diagnosis to treatment (54.9 days vs. 39.2 days, *p* = 0.044).

**Table 8:** Treatment timeline for outpatient diagnosis by multidisciplinary clinic enrollment

	Underrepresented minorities (URM)	Non-URM	Significance
*Non- pancreatic multidisciplinary clinic (PMDC) patients—outpatient*			
Time to Treatment, Days (Mean)			
Presentation to diagnosis	36.25	17.95	***p* = 0.007**
Diagnosis to treatment	41.81	29.44	*p* = 0.153
Total time to treatment	78.06	47.39	***p* = 0.006**
Time to treatment, Days (Median)			
Presentation to diagnosis	27.50	11.00	
Diagnosis to treatment	21.50	20.00	
Total time to treatment	49.00	40.00	
*PMDC patients—outpatient*			
Time to treatment, Days (Mean)			
Presentation to diagnosis	14.38	16.52	*p* = 0.702
Diagnosis to treatment	40.56	22.68	***p* = 0.005**
Total time to treatment	54.94	39.19	***p* = 0.044**
Time to treatment, days (Median)			
Presentation to diagnosis	7.50	11.00	
Diagnosis to treatment	35.50	20.00	
Total time to treatment	49.00	34.00	

Bolded *p* values indicate statistical significance

All patients diagnosed with Pancreatic Ductal Adenocarcinoma in Outpatient Setting were included in this analysis. Data presented as Days

## Discussion

The impact of diagnosis setting on the timing of definitive treatment and overall survival has not been well established in pancreatic cancer. To our knowledge, this is the first and largest evaluation of its type in an integrated healthcare network that treats a diverse population of patients. We found that URMs were 1.2 times more likely to be diagnosed with PDAC during an EP. Studies have shown that patients diagnosed with cancer in the emergency department have an increased mortality.^13–16^ A study by Newsom-Davis et al. reported that African Americans were more likely to present through the Emergency Department and that EP patients with lung cancer had a fourfold higher risk of dying within the first month of diagnosis versus their outpatient counterparts even when standardizing for age, stage, and histological subtype.^16^ A subset analysis of our own URM population similarly found that the increase in the proportion of EP diagnoses was largely driven by the Black population. In addition to diagnosis location, it has been reported that delays in diagnosis of pancreatic cancer can lead to a poorer prognosis.^17^ Our initial results showed that regardless of diagnosis location, URM had significantly longer times from presentation to biopsy and definitive treatment. This delay in time to definitive treatment resulted in an average time to initiation of therapy that was >6 weeks, which has been shown to be suboptimal when treating PDAC.^6^ One meta-analysis included 34 studies from 2000–2020 and determined that even a 4-week delay resulted in decreased survival outcomes across seven different cancers.^18^ For these reasons, identifying populations at increased risk for delay in cancer diagnosis and definitive treatment is imperative to improving mortality and survival outcomes in pancreatic cancer.

Our study found that URMs took on average three times longer to receive a biopsy following initial presentation in the EP cohort and 1 week longer in the OP cohort compared with their non-URM counterparts. Although concerning, these findings are well-established in cancer care.^19^ Recent studies have shown that factors, such as systemic racism, access to quality healthcare, nonprivate insurance status, health literacy, cultural and psychosocial factors, as well as other global healthcare system factors, lead to delays in biopsies and diagnosis of breast cancer in Black females.^20–23^ Our group posits that given the significant discrepancy in PCP rates amongst the groups, a leading cause for delay in the URM EP cohort may be the lack of an appropriate point of access to the healthcare system and a subsequent increase in emergency department usage as their primary point of care.^24–29^ Additionally, our study showed that URM were not significantly more likely to present with symptoms, such as jaundice, abdominal pain, weight loss, or receive a biliary stent. Therefore, if URM patients are presenting disproportionately to the ED but are not sick enough to be admitted, then they also would be likely to be discharged and become responsible for coordinating their own care without PCP support. This could result in patients being required to navigate the outpatient healthcare system to receive diagnostic biopsies. This idea is highlighted by our finding that URM who presented to the ED and were not admitted to the hospital following EP took on average 4 weeks longer to receive a biopsy following presentation than their admitted URM counterparts. It is important to note that the patients represented in those numbers, albeit a small sample size, are the ones who continued with care in the system and received a biopsy. This does not capture the patients who were discharged from the ED and were lost to follow-up and likely underestimates this disparity. Challenges to this process, such as poor health literacy, decreased trust in the healthcare system and limited resources for URM have been reported extensively and could be key contributors to our observed delay.^30^ Interestingly, we also found that older age was associated with increased time between biopsy and treatment and overall time to treatment in all patients in the outpatient setting. This finding could explain yet again the difficulties associated with navigating the healthcare system in the outpatient setting for certain patient populations. Perhaps retaining “at-risk” patients for expedited inpatient biopsy may mitigate some of these disparities.

Biopsy and treatment delays also occurred in the outpatient setting and may result from a combination of factors. One such factor is a decreased rate of URMs being evaluated by surgeons for resectable pancreatic tumors. A study by Riall et al. determined that Black patients were substantially less likely to undergo surgical evaluation which could be an explanation for delayed time to treatment in patients with PDAC.^31^ A recent study by Jager et al. determined 125 potential disparity-sensitive surgical measures that can provide concrete metrics to be followed with the intent on improving equity of surgical care in PDAC patients.^32^ Utilization of such measures could be beneficial to eliminating our observed delay in treatment time. In addition to decreased surgical evaluation, research has shown that underserved patient populations who are more likely to have Medicaid have limited access to physicians and experience increased wait times in the primary care setting compared with commercially insured patients.^33,34^ These findings could explain the increased number of URM patients presenting initially to the ED as well as the observed delays in time to biopsy and treatment in the outpatient setting. Alongside decreased evaluation and increased wait times, research has shown that minority patients pain symptoms are less likely to be addressed adequately due to implicit bias and incorrect beliefs about biological differences in healthcare workers leading to decreased diagnostic accuracy.^35^ Importantly, Charlot et al. showed in 2022 that utilization of their Accountability for Cancer Care through Undoing Racism and Equity (ACCURE) antiracism intervention, which included racial equity training, improved patient system navigation and race-specific reporting on treatment completion rates to clinical teams, was able to improve time to surgery in black lung cancer patients.^36^ When developing strategies to decrease URMs time to biopsy and treatment in the outpatient setting these factors must be addressed and routine implementation of antiracism interventions should be adopted where possible to overcome barriers negatively impacting patient care in this underserved patient population.

Delayed times to diagnosis and treatment in URMs may be due to racial barriers impacting their ability to navigate outpatient healthcare systems and PMDC inclusion improved discrepancies in time to treatment. Enrollment in our PMDC likely increases URMs access to care networks outside of the hospital, which may be lacking for discharged URMs without PCPs.^37,38^ That being said, URMs with PCPs were still more likely to present emergently and have delayed times from presentation to biopsy and definitive treatment. These results suggest there is something underlying the care provided for these patients that is slowing down their diagnosis and treatment. Additional barriers to biopsy acquirement and definitive treatment could be URMs access to healthcare due to location and social determinants of health. URMs possessing decreased health literacy may be less likely to seek medical assistance when necessary or have an inability to physically travel to tertiary care centers due to transportation or economics. Both obstacles could lead to increased times to treatment.^39^ It is important to note that our PMDC is primarily based in one region, and full expansion to other regions may better address access issues. While centralization of cancer care improves overall survival in pancreatic cancer, it does not always lead to increased access and utilization of care for minorities.^40,41^ Future efforts must highlight the need for healthcare systems to identify URM patients at increased risk for delayed times to diagnosis and treatment. One follow-up to this study might be to fully understand the denominator of new pancreas cancer diagnoses to elucidate the retention rate of URMs and identify areas to increase matriculation to definitive cancer care. Creating innovative solutions could include such policies as integrating our PMDC program into the inpatient hospital setting and emergency department to ensure patients who present emergently and have positive imaging for PDAC are not discharged unless a biopsy has been performed. By streamlining care in the hospital setting, URM who are more likely to present via this route could benefit greatly.

### Limitations

There are several limitations to this study. First, this is a retrospective cohort study, and certain variables were incomplete in the database. Second, patients’ courses from initial presentation to ultimate diagnosis and treatment for PDAC are highly variable and unique to each case. The nuance of each case interpretation and issues leading to differences in diagnosis and treatment time cannot always be reflected in the larger scale data compilation. Additionally, the sample size of this study was limited because of factors, such as initial presentation to an outside institution, patients receiving second opinions from other healthcare systems, and patients lost to follow-up, all of which excluded patients from our analysis. Another limitation was the use of a confirmatory biopsy as the date of diagnosis. Management of PDAC often can be determined based off positive imaging results; thus, patients whose management decisions are decided following positive imaging were not reflected in this analysis, which may have distorted time to diagnosis and treatment compared with the general population. Additionally, sometimes multiple endoscopic ultrasound-guided needle biopsies were required to collect an adequate tissue sample, which could have distorted time variables in select patients. An important point to note is that the improvement in pancreatic cancer care and evolution of our healthcare system’s cancer institute is not reflected in patients evaluated before the introduction of the PMDC in 2018, which could lead to increased times to biopsy and treatment that are not reflective of current practices. Another limitation to our analysis was lack of stratification based on insurance status. Unfortunately, our healthcare system does not readily categorize patient insurances into subtypes, but future efforts will focus on uncovering insurance status’ impact on delays to cancer treatment. Lastly, although this study used a healthcare system representative of a diverse patient population, data was obtained from only one institution, thereby limiting its generalizability. Future efforts should focus on a multicenter, prospective review that measures variables discussed in this study in a control group against an antiracism initiatives group, such as seen in recent literature.

## Conclusions

Underrepresented minorities were more likely to be diagnosed via an emergency presentation and showed delayed times to biopsy and treatment as compared to their White, Asian, and non-Hispanic or Latino counterparts. Future studies are required to elucidate the specific factors that resulted in these findings to identify solutions that can decrease this observed bias.

## References

[1] Puckett Y, Garfield K. 2023 Pancreatic cancer. In: *StatPearls.* Treasure Island, FL. 30085538

[2] Salom F, Prat F (2022). Current role of endoscopic ultrasound in the diagnosis and management of pancreatic cancer. World J Gastrointest Endosc..

[3] Orth M (2019). Pancreatic ductal adenocarcinoma: Biological hallmarks, current status, and future perspectives of combined modality treatment approaches. Radiat Oncol..

[4] Khorana AA (2019). Time to initial cancer treatment in the United States and association with survival over time: an observational study. PLoS One..

[5] Luchini C, Capelli P, Scarpa A (2016). Pancreatic ductal adenocarcinoma and its variants. Surg Pathol Clin..

[6] Gamboa AC (2020). Optimal timing and treatment strategy for pancreatic cancer. J Surg Oncol..

[7] Riner AN (2020). Disparities in pancreatic ductal adenocarcinoma: The significance of hispanic ethnicity, subgroup analysis, and treatment facility on clinical outcomes. Cancer Med..

[8] Nipp R (2018). Disparities in cancer outcomes across age, sex, and race/ethnicity among patients with pancreatic cancer. Cancer Med..

[9] Lutfi W, Zenati MS, Zureikat AH, Zeh HJ, Hogg ME (2018). Health disparities impact expected treatment of pancreatic ductal adenocarcinoma nationally. Ann Surg Oncol..

[10] Tavakkoli A (2020). Racial disparities and trends in pancreatic cancer incidence and mortality in the United States. Clin Gastroenterol Hepatol..

[11] Moaven O (2019). Healthcare disparities in outcomes of patients with resectable pancreatic cancer. Am J Surg..

[12] Health N (2023). Northwell Health.

[13] Weithorn D (2020). Diagnosis setting and colorectal cancer outcomes: the impact of cancer diagnosis in the emergency department. J Surg Res.

[14] Delamare Fauvel A (2023). Diagnosis of cancer in the emergency department: a scoping review. Cancer Med..

[15] Solsky I (2018). Gastric cancer diagnosis after presentation to the ED: The independent association of presenting location and outcomes. Am J Surg..

[16] Newsom-Davis T (2017). The route to diagnosis: emergency presentation of lung cancer. Lung Cancer Manag..

[17] Gobbi PG (2013). The prognostic role of time to diagnosis and presenting symptoms in patients with pancreatic cancer. Cancer Epidemiol..

[18] Hanna TP (2020). Mortality due to cancer treatment delay: systematic review and meta-analysis. BMJ..

[19] Buac NP (2022). Disparities in patient and system factors explain racial/ethnic disparities in delayed time to treatment in muscle invasive bladder cancer. Urol Oncol..

[20] Lawson MB (2022). Multilevel factors associated with time to biopsy after abnormal screening mammography results by race and ethnicity. JAMA Oncol.

[21] George P (2015). Diagnosis and surgical delays in African American and white women with early-stage breast cancer. J Womens Health (Larchmt)..

[22] Reeder-Hayes KE (2019). Race and delays in breast cancer treatment across the care continuum in the Carolina Breast Cancer Study. Cancer..

[23] Schermerhorn MC, Grunvald MW, O'Donoghue CM, Rao RD, Becerra AZ (2022). Factors mediating racial/ethnic disparities in delayed treatment of breast cancer. Ann Surg Oncol..

[24] Nelson A (2002). Unequal treatment: confronting racial and ethnic disparities in health care. J Natl Med Assoc.

[25] Heron SL, Stettner E, Haley LL (2006). Racial and ethnic disparities in the emergency department: a public health perspective. Emerg Med Clin North Am..

[26] Zhang X (2020). Trends of racial/ethnic differences in emergency department care outcomes among adults in the United States from 2005 to 2016. Front Med (Lausanne)..

[27] Cone DC, Richardson LD, Todd KH, Betancourt JR, Lowe RA (2003). Health care disparities in emergency medicine. Acad Emerg Med..

[28] Fiscella K, Sanders MR (2016). Racial and ethnic disparities in the quality of health care. Annu Rev Public Health..

[29] Laiyemo AO (2010). Race and colorectal cancer disparities: healthcare utilization vs different cancer susceptibilities. J Natl Cancer Inst..

[30] Muvuka B (2020). Health Literacy in African-American communities: barriers and strategies. Health Lit Res Pract..

[31] Riall TS, Townsend CM, Kuo YF, Freeman JL, Goodwin JS (2010). Dissecting racial disparities in the treatment of patients with locoregional pancreatic cancer: a 2-step process. Cancer..

[32] de Jager E (2023). Disparity-sensitive measures in surgical care: a Delphi panel consensus. J Am Coll Surg..

[33] Gotlieb EG, Rhodes KV, Candon MK (2021). Disparities in primary care wait times in Medicaid versus commercial insurance. J Am Board Fam Med..

[34] Greene J, Blustein J, Weitzman BC (2006). Race, segregation, and physicians' participation in medicaid. Milbank Q..

[35] Hoffman KM, Trawalter S, Axt JR, Oliver MN (2016). Racial bias in pain assessment and treatment recommendations, and false beliefs about biological differences between blacks and whites. Proc Natl Acad Sci U S A..

[36] Charlot M (2022). Effect of an antiracism intervention on racial disparities in time to lung cancer surgery. J Clin Oncol..

[37] Hoehn RS (2021). A pancreatic cancer multidisciplinary clinic eliminates socioeconomic disparities in treatment and improves survival. Ann Surg Oncol..

[38] Khan H (2022). Fragmentation of care in pancreatic cancer: effects on receipt of care and survival. J Gastrointest Surg..

[39] Rose J (2022). Factors affecting timely breast cancer treatment among black women in a high-risk urban community: a qualitative study. BMC Womens Health..

[40] Hsu DS (2022). Centralization of pancreatic cancer treatment within an integrated healthcare system improves overall survival. Am J Surg..

[41] Lieberman-Cribbin W, Liu B, Leoncini E, Flores R, Taioli E (2017). Temporal trends in centralization and racial disparities in utilization of high-volume hospitals for lung cancer surgery. Medicine (Baltimore)..

